# Model-based cost-effectiveness analysis of the diagnosis and treatment of cow's milk protein allergy with amino acid-based formula compared to extensively hydrolyzed formula in Argentina

**DOI:** 10.3389/fped.2025.1543811

**Published:** 2025-05-08

**Authors:** Christian Boggio Marzet, Pablo Malagrino, Paula Micone, Norberto Giglio

**Affiliations:** ^1^ Grupo de Trabajo en Gastroenterología y Nutrición Pediátrica, Hospital Pirovano, Buenos Aires, Argentina; ^2^ Sección Gastroenterología, Hospital de Niños Dr Ricardo Gutiérrez, Buenos Aires, Argentina; ^3^ Departamento de Tocoginecología, Hospital Carlos G Durand, Buenos Aires, Argentina; ^4^ Epidemiología, Hospital de Niños Dr Ricardo Gutiérrez, Buenos Aires, Argentina

**Keywords:** cost-effectiveness evaluation, cow's milk allergy, extensively hydrolized formula, amino acids formula, cost—benefit analysis, cow's milk protein allergy

## Abstract

**Introduction:**

Cow's milk protein allergy (CMPA) is the most common food allergy in children under one year of age. The CMPA has a significant economic impact on health resources. The objective of this study was to estimate the cost-effectiveness of implementing a new diagnostic and treatment strategy using an amino acid-based formula in infants with suspected CMPA.

**Materials and methods:**

A simple decision tree was developed. The model simulates a cohort of Argentine children of less than 6 months with suspected CMPA who were followed with clinical checks until they were 24 months of age. The first arm considers the standard of care for diagnosis and treatment of children with suspected CMPA that suggest eliminating whole cow's milk proteins and initiating treatment with (extensively hydrolyzed formula (eHF). A diagnostic process time of 4 weeks was estimated. The second arm investigates the impact of a new diagnosis and treatment strategy that eliminates cow's milk proteins and prescribes an elementary amino acid-based formula (AAF). A period of 4 weeks was estimated to assess the diagnosis of CMPA.

**Results:**

Using an AAF for the diagnosis and treatment of a cohort of 12,334 children with suspected CMPA, less six month age, resulted in a saving of 3,368,176 usd and 334 months gained without symptoms,. The use of AAF, as a first line treatment, was cost saving. These results proved to be robust in the one-way sensitivity analysis.

**Conclusions:**

A diagnostic strategy using AAF offers cost savings and reduces the duration of the symptomatic period, allowing effective treatment to be established earlier, which in turn reduces direct medical expenses.

## Introduction and objective

Cow's milk protein allergy (CMPA) is the most common food allergy in children under one year of age (1–3).

It is an immune-mediated reaction caused by the proteins in cow's milk. Despite the existence of different guidelines and recommendations in the management of children with CMPA, there is great variability in their diagnosis and treatment, especially in non-IgE-mediated forms.

A detailed medical chart and challenge-exclusion test are the only tools available to diagnose this type of non-IgE-mediated allergy.

The incidence in the first year of life is estimated to be 2%–3% (3). In a study conducted in Chile in 2014, the incidence found was 4.9% in children under 12 months of age (4).

The CMPA has a significant economic impact on public and private health resources. However, only a few publications have evaluated its diagnostic and therapeutic management from a financial perspective (5).

This pediatric disease diagnosis and treatment standard requires a period based on the elimination of cow's milk proteins, by: prescribing formulas with extensively hydrolyzed cow's milk proteins (eHF) together with the exclusion of cow's milk proteins from the maternal diet, unless symptoms suggestive of CMPA had started after incorporating infant formula (6, 7).

In any case, the diagnostic process is followed by a challenge test with formulas containing cow's milk protein after the symptomatic improvement (8, 9).

Extensively hydrolyzed formulas are hypoallergenic. However, they are not completely allergen-free, so complete recovery from allergic symptoms is sometimes not achieved in all patients. It is estimated that symptoms will not resolve in 5%–10% of patients with CMPA with an eHF (7, 8). In these cases, changing these formulas to those containing only amino acids will be necessary to mitigate CMPA symptoms in 100% of cases (10, 11).

Based on this rationale, it is logical to consider that amino acid-based formulas (AAF) promote and accelerate clinical improvement in all patients with CMPA, in addition to confirming the diagnosis of CMPA earlier in patients who do not respond to therapy with extensively hydrolyzed formulas.

However, due to the higher cost of amino acid-based formulas over extensively hydrolyzed formulas, the former are used for more severe cases or for patients who do not tolerate extensively hydrolyzed formulas.

The objective of this study was to estimate the cost-effectiveness of implementing a new diagnostic and treatment strategy using an amino acid-based formula instead of an extensively hydrolyzed formula in infants with suspected CMPA.

## Material and methods

A simple decision tree model was developed using TreeAgePro 2021 (TreeAge Software, Inc., Williamstown, MA). In this regard, the benefits and costs of two diagnostic and treatment strategies for CMPA were estimated.

**Figure 1:**
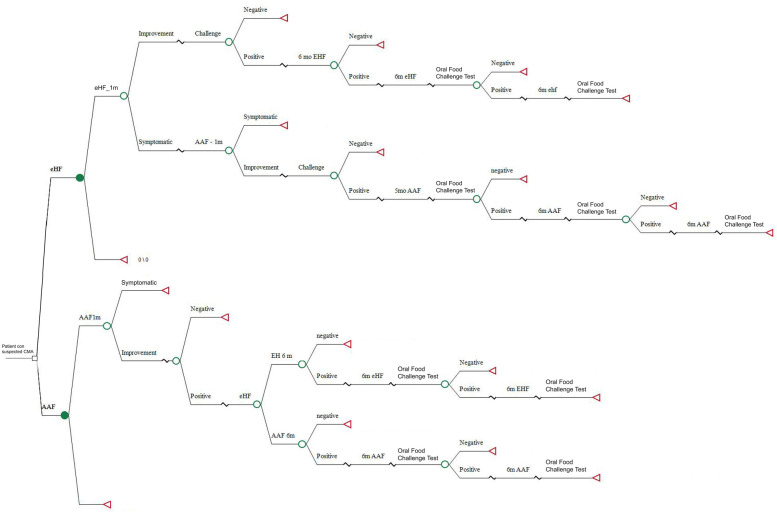
Simple decision tree model.

A simple decision tree was developed, a tool accepted by the scientific community and regulatory agencies as one of the standard modeling platforms. The model simulates a cohort of Argentine children of less than 6 months with suspected CMPA who were followed with clinical checks until they were 24 months of age (Figure 1).

The first arm considers the standard of care for diagnosis and treatment of children with suspected CMPA according to national and international recommendations that suggest eliminating whole cow's milk proteins and initiating treatment with eHF. To be in line with general recommendations, a diagnostic process time of 4 weeks was estimated by feeding extensively hydrolyzed protein formula before the challenge test. Patients who continued with symptoms despite using extensively hydrolyzed protein formula were prescribed an amino acid-based formula.

**Figure 2:**
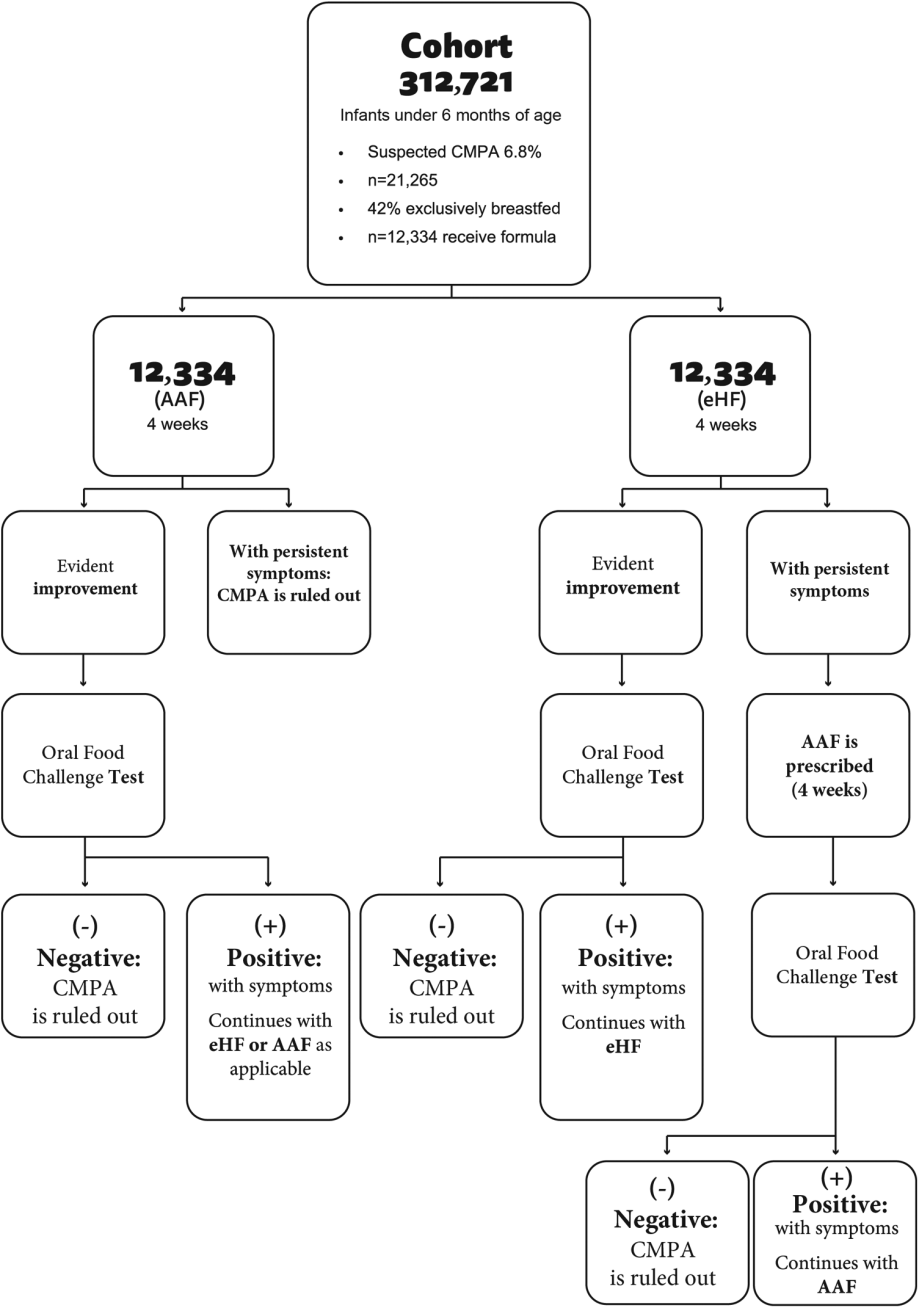
Decision tree according to different diagnostic strategies for CMPA.

The second arm investigates the impact of a new diagnosis and treatment strategy that eliminates cow's milk proteins and prescribes an elementary amino acid-based formula. A period of 4 weeks was estimated to assess the diagnosis of CMPA. In this case, if the challenge test is negative, CMPA is ruled out and the patient is excluded from the study. If the test is positive, the diagnosis of CMPA is confirmed (Figure 2).

In cases confirmed after the challenge test, children are exposed to new challenge tests every 6 months until they are 24 months of age. Formula prices were obtained according to market cost values (12). USD was used at the 2024 exchange rate.

The cohort excluded children who were exclusively breastfed (42%) (13).

An estimated 6.8% of children younger than 6 months have symptoms compatible with CMPA, while the prevalence of confirmed cases was estimated at 2% (Table 1).

**Table 1:** Data entered into the cost-effectiveness economic model.

Variables	Point estimate	References
Children of less than 6 months, Argentina, 2019	3,12,720	(15)
Incidence of Suspected CMPA	6.8%	(5)
Incidence of CMPA	2%	(16)
eHF Effectiveness	90%	(5)
AAF Effectiveness	100%	(5)
Exclusively breastfed	42%	(15)
Mixed	46%	(15)
Tolerance to cow's milk at 1 year old	56%	(5)
Tolerance to cow's milk at 2 years old	77%	(5)
AAF cost/month USD 9 months old	739	
AAF cost/month USD 6 months old	1,426	
eHF cost/month USD 9 months old	673	
eHF cost/month USD 6 months old	1,170	

The calculation of monthly formula consumption was based on the requirements of a 6-month-old male infant, 50th percentile of weight, who was exclusively fed with formula and then at 9 months of age, considering that he would receive 50% of the nutritional intake from the formula. Since the study was conducted from the healthcare provider's perspective, the following equations were considered for formula consumption:

Patient aged 6 months old exclusively formula-fed:
Monthly energy consumption at 6 months = 50th perc weight male × kcal/kg/day (14) × 30 days.
Monthly energy consumption at 6 months = 8 kg × 78 kcal/kg/day × 30 days = 18,720 kcal
Number of monthly cans = Monthly kilocalories/kilocalories of 1,400 g can
Monthly number of cans = 18,720 kcal/1,864 kcal = 10 cans
Patient aged 9 months old with 50% formula requirements
Monthly energy consumption at 9 months: 50th perc weight males × kcal/kg/day (14) × 30
Monthly energy consumption = 9 kg × 77 kcal/kg/day × 30 days = 20,790 kcal
Number of monthly cans = Kcal monthly/kcal of 1 can of formula 400 g × 0.5
Monthly number of cans = 20,790/1,864 × 0.5 = 6 cans

A 6-month-old patient was considered to be exclusively formula-fed while a 9-month-old patient was considered to have 50% formula requirements.

**Figure 3:**
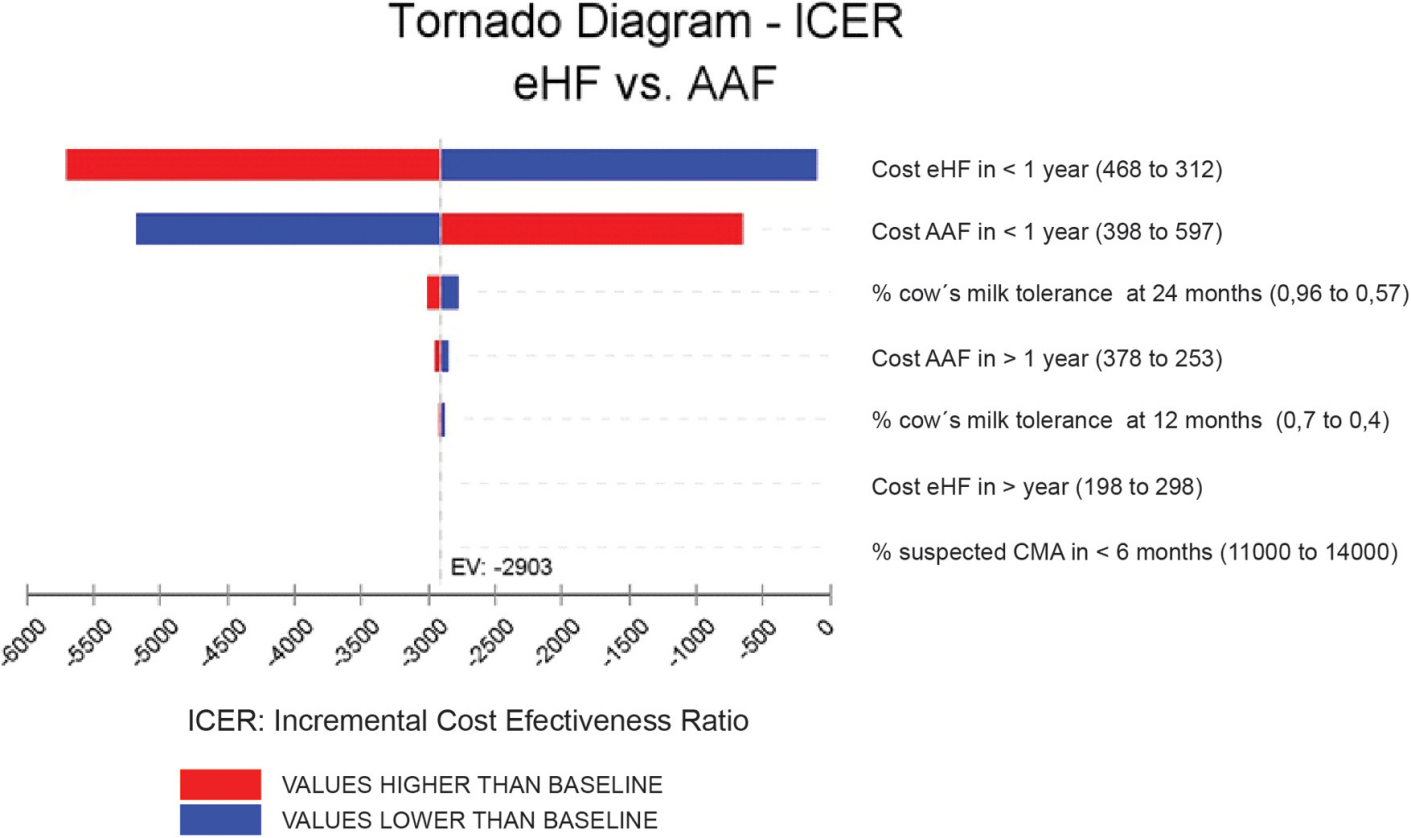
Tornado diagram.

Results were expressed as incremental cost per month of symptom-free living gained over a 24-month time frame. To address the uncertainty, a sensitivity analysis was performed with a tornado diagram understanding that the tornado has the extreme values for the variables entered into the model (Figure 3).

## Results

A cohort of 312,721 Argentine children under 6 months of age was simulated. It was assumed that 42% were exclusively breastfed (131,342) and that 6.8% will have symptoms compatible with CMPA. Therefore, in this model, 12,334 children will be annually exposed to an elimination diet followed by a challenge test.

Table 2 shows the clinical and economic results of both strategies. Using an AAF for the diagnosis and treatment of children with suspected CMPA resulted in more benefits and fewer costs (dominated strategy).

**Table 2:** Cost-effectiveness analysis.

Formula	Cost in USD	Avoided cost	Months without symptoms	Months without symptoms gained	ICER
AAF	56,564,020	0	51,941	0	
eHF	59,932,197	3,368,176	51,607	−334	Dominated

ICER, incremental cost-effectiveness ratio; AAF, amino acid-based formulas; eHF, extensively hydrolyzed protein formulas.

**Table 3:** One-way sensitivity analysis.

Variable Name	Variable Low	Variable Base	Variable High	Low	High
Monthly cost of eHF in infants under 1 year of age	936	1,170	1,404	Dominated	Dominated
Monthly cost of AAF in infants under 1 year of age	1,140	1,426	1,711	Dominated	Dominated
Monthly cost of AAF in infants older than 1 year of age	591	739	886	Dominated	Dominated
Monthly Cost of eHF in infants older than 1 year of age	539	673	808	Dominated	Dominated
Tolerance to cow's milk at 24 months of age	0.57	0.77	0.96	Dominated	Dominated
Tolerance to cow's milk at 12 months of age	0.4	0.56	0.7	Dominated	Dominated
6 months of age with CMPA-like symptoms	11,000	12,333	14,000	Dominated	Dominated

To assess the uncertainty of the model, a tornado diagram was developed (see Annexes, Figure 3). Variations in ICER are shown, assuming a ±25% variability in each variable. The two variables with the greatest impact on ICER were the costs of each formula for the cohorts. However, it is observed that, even using the highest value for AAF and the lowest value for eHF, the results continue to show the dominance of the new strategy compared to the standard one. It is considered a dominated strategy when the health benefits are greater for this treatment and when the costs of the strategy are lower (Table 3).

## Discussion

The prevalence of food allergy is increasing, with CMPA being the most common one in children under 3 years of age (17–19).

To our knowledge, this study is the first in Spanish-speaking Latin American countries to evaluate the cost-effectiveness of an approach other than conventional CMPA treatment (6, 9). It is a theoretical model based on data obtained from the world literature with national demographic data (13, 15).

Our study poses the initial use of an AAF as a therapeutic setting to quickly exclude non-allergic patients who continue with symptoms for other reasons or who do not have symptoms with the challenge test (19).

After the diagnostic period, patients with confirmed CMPA diagnosis can continue with eHF. The highest percentage will remain asymptomatic with eHF until tolerance is acquired. Finally, a percentage of these allergic patients will need to return to an elementary AAF, since as previously discussed, 10% of patients with CMPA do not respond to eHF (5).

Treating cow's milk allergy in children is expensive for families (20). In Argentina, according to Law No. 27305, the Argentine National State and its health systems provide treatment at no cost (21), a condition that “alleviates” out-of-pocket family expenses, but in terms of budget, public expenses increase because these formulas must be provided.

The work of Cawood and collaborators demonstrates that the economic impact of children with CMPA is associated with a series of comorbidities that are added to children suffering from this disease. In this regard, children with food allergies have other comorbidities that also require repeated visits to the pediatrician, gastroenterologist or allergist and increase indirect costs, such as increased absenteeism from work and the higher cost of a special diet (22).

In our study, we have not taken into account these indirect costs or out-of-pocket expenses considering that the expense of diagnosing and treating CMPA is mostly focused on the cost of AAF or eHF.

Pharmacoeconomic results show that introducing an AAF at diagnosis is cost-saving, understanding this definition where a strategy is cheaper and more effective.

It is worth highlighting that our results match those reported by Morais et al. who performed a similar strategy in a Brazilian population (5).

Beyond this economic benefit in global terms, it was concluded that the use of an AAF at the diagnostic stage decreases the symptomatic days of the population. The absence of symptoms would mean a reduced need for access to health equipment and complementary medical studies. In this regard, it is worth stressing that our study was conducted with a conservative approach that considered only direct medical costs. In this approach, our work may be underestimating the additional economic benefits. However, two points have not yet been answered. We can also consider whether it is actually plausible in practice that those patients who show improvement with AAF will later switch to eHF knowing that there is a (remote) possibility that symptoms may recur. Concerning the timing of tolerance acquisition, what is the difference between AAF or eHF in IgE-mediated and non-IgE mediated allergies? Further studies will probably be required to answer these last points.

Our study presents strengths and weaknesses. As for its strengths, this is the first pharmacoeconomic analysis carried out in Argentina with local costs. As a weakness, not considering secondary costs will surely be underestimating the benefits of this alternative strategy. In any case, the pharmacoeconomic approaches of this study will be helpful for decision-making. In real-world settings, the involvement of patients or other external actors not considered in the model—such as caregiver adherence, prescribing habits, or socioeconomic barriers—could introduce variability not shown in our decision tree. These factors may influence treatment adherence and symptom reporting, potentially affecting the perceived cost-effectiveness. While our model reflects standardized clinical pathways, we recognize that real-world dynamics could impact decision-making and should be considered when interpreting results. That`s why, even if the model results are strong, it is crucial to monitor and adjust the implementation according to each real-life setting.

## Conclusion

A diagnostic strategy using AAF offers cost savings and reduces the duration of the symptomatic period, allowing effective treatment to be established earlier, which in turn reduces direct medical expenses.

## Data Availability

The original contributions presented in the study are included in the article/Supplementary Material, further inquiries can be directed to the corresponding author.
